# ERP Subsequent Memory Effects Differ between Inter-Item and Unitization Encoding Tasks

**DOI:** 10.3389/fnhum.2017.00030

**Published:** 2017-01-30

**Authors:** Siri-Maria Kamp, Regine Bader, Axel Mecklinger

**Affiliations:** Department of Psychology, Saarland UniversitySaarbrücken, Germany

**Keywords:** subsequent memory effect, event-related potentials, P300, frontal slow wave, episodic memory

## Abstract

The “subsequent memory paradigm” is an analysis tool to identify brain activity elicited during episodic encoding that is associated with successful subsequent retrieval. Two commonly observed event-related potential “subsequent memory effects” (SMEs) are the parietal SME in the P300 time window and the frontal slow wave SME, but to date a clear characterization of the circumstances under which each SME is observed is missing. To test the hypothesis that the parietal SME occurs when aspects of an experience are unitized into a single item representation, while inter-item associative encoding is reflected in the frontal slow wave effect, participants were assigned to one of two conditions that emphasized one of the encoding types under otherwise matched study phases of a recognition memory experiment. Word pairs were presented either in the context of a definition that allowed to combine the word pairs into a new concept (unitization or item encoding) or together with a sentence frame (inter-item encoding). Performance on the recognition test did not differ between the groups. The parietal SME was only found in the definition group, supporting the idea that this SME occurs when the components of an association are integrated in a unitized item representation. An early prefrontal negativity also exhibited an SME only in this group, suggesting that the formation of novel units occurs through interactions of multiple brain areas. The frontal slow wave SME was pronounced in both groups and may thus reflect processes generally involved in encoding of associations. Our results provide evidence for a partial dissociation of the eliciting conditions of the two types of SMEs and therefore provide a tool for future studies to characterize the different types of episodic encoding.

## Introduction

A widely used approach to study the neuro-cognitive basis of episodic encoding is to compare physiological activity elicited by to-be-encoded stimuli between those that are retrieved on a subsequent memory test, vs. those that are not. While this “subsequent memory paradigm” (for reviews, see Wagner et al., 1999; Paller and Wagner, 2002; Werkle-Bergner et al., 2006) was first applied to event-related potentials (ERPs; Sanquist et al., 1980; Karis et al., 1984), recently the majority of studies have used functional magnetic resonance imaging (fMRI). Perhaps part of the receding enthusiasm for conducting subsequent memory studies with ERPs is due to the large between-study variance in the ERP components that exhibit “subsequent memory effects” (SMEs). Some studies have even failed to find any SMEs at all (Johnson, 1995), which may lead some to conclude that ERP SMEs are unreliable. The present study was designed to resolve some of these inconsistencies by characterizing the circumstances under which two SMEs with specific spatio-temporal characteristics occur.

### Different Types of ERP Subsequent Memory Effects

Perhaps the most frequently reported ERP SMEs are found (1) at parietal electrodes, exhibiting spatio-temporal characteristics consistent with the P300, and (2) at mostly frontal electrodes as modulation of ERP slow waves. In the present study we examine the idea that unitization, as a special case of encoding in which two items are processed as a single coherent unit, and inter-item associative encoding, are differentially associated with these two SMEs. It should be noted that ERP SMEs with different spatio-temporal characteristics are also dissociable according to other dimensions, such as the nature of the study material (e.g., Wagner et al., 1999), and can exhibit different time courses with onsets sometimes even before stimulus presentation (e.g., Gruber and Otten, 2010). Our focus, however, is on the item vs. inter-item associative encoding distinction of stimulus-elicited SMEs. The next two sections will briefly review prior studies that have reported one of the two SMEs under consideration.

#### Parietal SME

A parietal SME has first been reported in paradigms designed to study the P300, a positivity that is large for task-relevant events that deviate from the individual’s expectancies (Donchin, 1981; Donchin and Coles, 1988). Such events are also more likely to be retrieved in subsequent free recall tests (Von Restorff, 1933). These two patterns are linked, such that larger P300 amplitudes are associated with those deviant events that are subsequently freely recalled, compared to those that are not (Karis et al., 1984; Fabiani et al., 1986, 1990; Fabiani and Donchin, 1995; Otten and Donchin, 2000; Wiswede et al., 2006; Kamp et al., 2013; Kamp and Donchin, 2015). However, the parietal SME is not always observed when stimuli are distinctive: It is absent when participants encode stimuli by focusing on relationships between items rather than features of the individual items (e.g., Karis et al., 1984; Fabiani et al., 1990). Notably, such strategies also abolish the behavioral recall enhancement for deviant items (e.g., Karis et al., 1984). Parietal (P300) SMEs have also been reported in free recall paradigms for the first word in a list (Azizian and Polich, 2007; Kamp et al., 2012), and for emotionally negative items (Kamp et al., 2015), which due to their unique serial position or emotional content may “stand out” just like items that violate expectancies. However, when an item is distinctive due to a non-integral feature (such as a frame around a word or a background picture), the P300 is not accompanied by a parietal SME (Otten and Donchin, 2000; Wiswede et al., 2006). Taken together, these patterns support the view that item, rather than contextual or associative, encoding is indexed by the parietal SME. That is, when participants encode information that renders items (or coherent units) distinctive and subsequently use (or are provided with) accordant retrieval cues, the parietal SME is observed.

All task-relevant stimuli – not only those that are deviant or salient – elicit at least a small P300. Perhaps it is therefore not surprising that SMEs with a similar time course that are maximal at parietal electrodes are also observed when the study phase of a memory experiment does not entail a manipulation of deviance. For example, Bauch and Otten (2012) found a parietal SME in a condition in which pictures of objects were encoded, but recognition for verbal labels of the objects was tested. Gonsalves and Paller (2000) instructed participants to form mental images of words during encoding. After some of the words, additionally a picture of the object was shown and at test, participants judged for each word whether a corresponding picture had been presented. Analogously to Bauch and Otten’s results, the pictures elicited a parietal SME. A similar effect was also elicited by words for which participants subsequently falsely indicated that they had seen a picture during encoding. Thus, when pictorial information is retrieved based on verbal probes, participants may search their memory for a representation containing rich visual, i.e., item-specific, information (regardless of whether these features are imagined or “real”). The result patterns are therefore consistent with the idea that when item-specific and distinctive features are encoded (and subsequently retrieved), the parietal SME is observed.

Taken together, the parietal SME seems to be large when the details of an item are effectively encoded (and subsequently utilized at retrieval), it is not modulated by contextual factors of the study situation and it is absent or weak when participants do not retrieve rich item-specific information at test. This leads to the hypothesis that in encoding conditions in which unitized item representations are formed, the parietal SME occurs.

#### Frontal Slow Wave SME

When participants use encoding strategies that emphasize relations between items rather than item-specific information an ERP component with a longer latency and a (typically) frontal distribution often shows an SME (Karis et al., 1984; Fabiani et al., 1990). When lists are encoded in this manner, retrieval may be cued by means of an association to other, already recalled items (e.g., Kahana, 1996), and therefore may rely less on item specific details. The process of creating and encoding flexible links between discrete stimuli is called *inter-item associative* encoding and it likely relies on simultaneous maintenance and manipulation of multiple items in working memory. In support of this, fMRI studies have shown that dorsolateral prefrontal cortex (DLPFC) activity, which reflects active working memory manipulations, co-varies with the success of inter-item associative encoding (Blumenfeld and Ranganath, 2006; Blumenfeld et al., 2011). This kind of working memory activity may be indexed by ERP slow waves, as they vary in amplitude and topography with both the type and the amount of information maintained in working memory (Ruchkin et al., 1990, 1991; Mecklinger and Pfeifer, 1996; Bosch et al., 2001; for reviews, see Johnson, 1995; Ruchkin et al., 2003), as well as with the number of associations that are retrieved from long-term memory (Khader et al., 2005).

If the frontal slow wave is an electrophysiological correlate of working memory processes that support the creation of inter-item associations, slow wave SMEs should be observed particularly in tasks in which item pairs are encoded and subsequently retrieved relationally. Indeed, for pairs of semantically meaningful study items, a slow wave SME with a frontal distribution occurs (e.g., Weyerts et al., 1997; Jäger et al., 2006; Kim et al., 2009; Kamp and Zimmer, 2015). This SME also appears to be larger for deep- than shallow encoding tasks (Guo et al., 2004). Based on these findings, we hypothesized that elaborate inter-item encoding is reflected by modulations of the frontal slow wave SME.

In the present study we thus tested the idea that the parietal SME occurs for item encoding, while the frontal slow wave SME occurs under conditions that promote the encoding and retrieval of inter-item associations. Kim et al. (2009) proposed a similar idea, but based their proposal on results from a paradigm where cued recall was tested. This test format relies on both item- and associative memory, so it does not allow for an unambiguous interpretation of the SMEs in this framework. Accordingly, based on a re-analysis of these data, Kim et al. (2012) arrived at the opposite conclusion that the parietal SME indexes associative, rather than item, encoding. Mecklinger and Müller (1996), too, have previously proposed that the slow wave SME occurs when inter-item processing is engaged. However, they only reported indirect, *post hoc* evidence based on post-experimental questionnaires. In order to disentangle item vs. inter-item associative encoding, a better paradigm would directly contrast two conditions that each emphasize only one or the other of the two encoding types under otherwise matched encoding conditions and stimulus materials.

### The Present Study

During a recognition test retrieval of inter-item associations typically requires a context-dependent effortful process known as *recollection*, while retrieval of individual items can occur via a context-free, automatic process called *familiarity* (Yonelinas, 2002). These modes of retrieval are reflected in distinct ERP effects elicited at retrieval: a late parietal old/new effect indexes recollection and an early frontal old/new effect indexes familiarity (for a review, see Rugg and Curran, 2007). Of note, there is increasing evidence that familiarity not only supports item recognition, but can also contribute to associative recognition, in particular in situations in which two items can be fused into a single unitized representation. This later view has been confirmed by a number of recent neuroimaging (e.g., Haskins et al., 2008), neuropsychological (e.g., Quamme et al., 2007), and ERP studies (e.g., Bader et al., 2010). For example, the paradigm used in the present study has been shown to elicit an early ERP old/new effect (Bader et al., 2010) in one condition, supporting the view that associations are encoded as unitized item representations.

**FIGURE 1:**
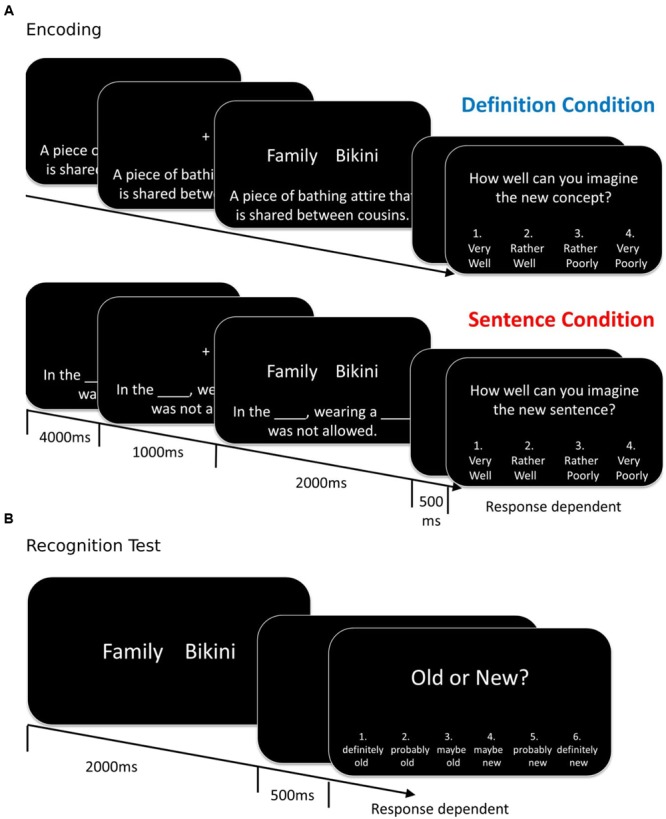
**Experimental design: Trial structure at encoding and recognition. (A)** Participants were randomly assigned to either the definition- or the sentence encoding condition. Encoding event-related potential (ERPs) were time locked to the onset of the word pair. **(B)** The test structure was identical for participants in both conditions. In both the encoding and test phase, the inter-trial interval (from the offset of the rating screen to the onset of the next sentence/definition or the next word pair, respectively) was 2000 ms. Example stimuli have been translated from German to English (The German word pair is “Familien, Bikini”; the first word is in the German version in plural form to allow for the pair to be a legal compound word. See main text for details).

We thus employed a paradigm recently used to study the effects of encoding conditions that promote unitization on associative recognition (Quamme et al., 2007; Haskins et al., 2008; Bader et al., 2010, 2014; Parks and Yonelinas, 2015): Participants encode word pairs either in the context of a definition that provides a basis to interpret the pair as a compound word, or in the context of a sentence frame that allows encoding of the words relationally, but maintains the representation of each word as an individual unit (see **Figure 1** for an example). A compound word with a new, joint meaning can be considered a single unit, and accordingly, the definition condition has also been termed the “unitization” condition (see also Graf and Schacter, 1989).

Consistently, studies using this paradigm have shown that retrieval of word pairs in the sentence condition elicits brain activity previously associated with recollection (Haskins et al., 2008; Bader et al., 2010, 2014; Kamp et al., 2016). In the definition (or unitization) condition, retrieval is accompanied by neural correlates of familiarity, which provides additional support for the view that word pairs in this condition were encoded as single units. Furthermore, in the definition condition only, fMRI activity in perirhinal cortex (PrC), which is usually associated with item encoding, predicts subsequent familiarity-based retrieval (Haskins et al., 2008). Thus, using this paradigm we disentangled the formation of a single item representation from inter-item associative encoding that retains each of the two words as individual units. At the same time, the present study allows us to investigate whether the dissociation between the activated brain regions in the definition (unitization) vs. sentence (inter-item) encoding condition observed by Haskins et al. (2008) would also be observable in ERP SMEs.

We thus analyzed ERPs in the encoding phase of a version of this paradigm that is suitable for ERP subsequent memory analyses. In the definition condition, item encoding processes should result in a pronounced parietal SME. Conversely, in the sentence condition, associative inter-item encoding of the two words without facilitating their integration into a unit should result in a frontal slow wave SME. A between-subject design was employed to prevent strategic carry-over effects between both encoding conditions and for the purpose of consistency with prior studies using the paradigm.

In order to disentangle ERP component overlap, in addition to a mean amplitude analysis we employed a spatio-temporal principal component analysis (PCA; Spencer et al., 1999) to extract factor scores as measures of ERP component amplitudes. For the sake of simplicity we present only combinations of spatial and temporal factors (TFs) that exhibited the typical characteristics of the SMEs under exploration in this study.

## Results

### Behavioral Data

**FIGURE 2:**
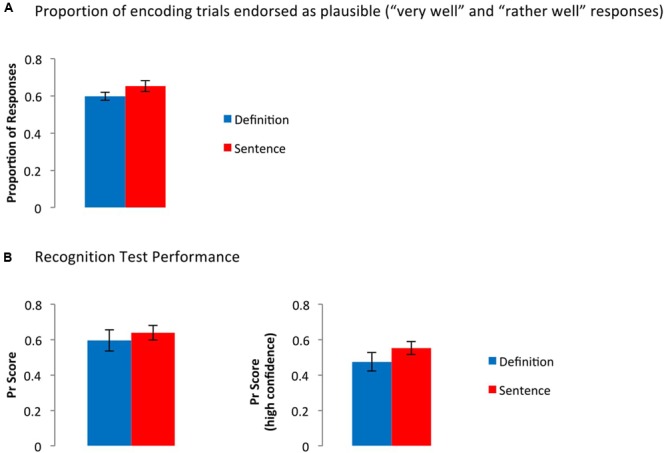
**Behavioral data from (A)** the encoding phase and **(B)** the recognition phase. **(A)** Frequency of trials rated as plausible, collapsed across levels 1 and 2 (“very well” and “rather well”). **(B)** Pr scores collapsed across confidence level (left panel), or only for high confidence responses (right panel). Error bars represent the standard error of the mean. *T*-tests between groups were not significant in any case.

There was no significant difference between groups in the probability that participants endorsed the new concepts or sentences as plausible at encoding (**Figure 2A**), *t*(33) = 1.56, *p* = 0.13. Recognition performance, too, was comparable between groups: Pr scores did not differ between groups, regardless of whether hits/false alarms across all confidence levels, *t*(33) = 0.61, *p* = 0.54, or only high confidence responses, *t*(33) = 1.23, *p* = 0.23, were considered (**Figure 2B**).^1^

### Event-Related Potential (ERPs)

**FIGURE 3:**
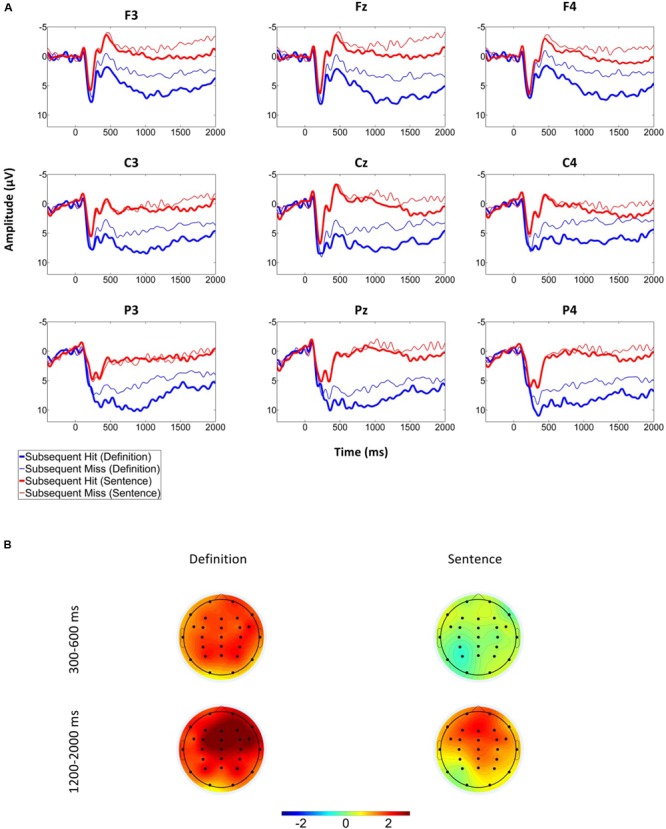
**Grand average ERPs for the sentence and definition groups depending on subsequent memory. “Subsequent Hit” trials are those that received a high confidence “old” response at test, while “Subsequent Miss” trials include all other trials.** See text for details. **(A)** Grand Averages from nine electrodes included in the analysis. **(B)** Difference of the mean amplitudes of subsequent hits and misses in the two time windows analyzed.

**Figure 3** suggests that the grand average ERPs elicited by the onset of the word pair at encoding were more positive-going in the definition than the sentence group and for subsequent hits than for subsequent misses. Furthermore, there was a difference between ERPs elicited by subsequent hits vs. misses at an earlier time point for the definition- (onset at about 300 ms) than the sentence (onset at about 1000 ms) group. The early SME in the ERPs elicited in the definition group appeared to be due to a larger positivity at parietal as well as an attenuated negativity at frontal electrodes for subsequent hits. Furthermore, in both conditions a frontally distributed slow wave SME was apparent in a late (>1000 ms) time window.

#### Mean Amplitude Analysis

We analyzed the mean amplitudes in the P300 and the slow wave time windows in 3 (anteriority: frontal, central, parietal) by 3 (laterality: left, mid, right) by 2 (subsequent memory: subsequent hit, subsequent miss) by 2 (group: definition, sentence) mixed ANOVAs. In all analyses there were main effects of group (all *p*s < 0.05): Amplitudes were more positive-going for the definition than the sentence group. In the following, we will only report main effects for or interactions with the factor subsequent memory.

##### P300 time window (300–600 ms)

There was a main effect of subsequent memory, *F*(1,33) = 5.21, *p* = 0.03, η_p_^2^ = 0.14, qualified by an interaction between subsequent memory and group, *F*(1,33) = 4.41, *p* = 0.04, η_p_^2^ = 0.12. Separate 3 (anteriority) by 3 (laterality) by 2 (subsequent memory) ANOVAs for each group revealed a main effect for subsequent memory for the definition, *F*(1,16) = 8.46, *p* < 0.01, η_p_^2^ = 0.35, but not for the sentence group, *F*(1,17) = 0.02, *p* > 0.89. There were no interactions of subsequent memory with any other factor for either group (all *p*s > 0.38).

##### Frontal slow wave time window (1200-2000 ms)

There was a main effect for subsequent memory, *F*(1,33) = 8.33, *p* < 0.01, η_p_^2^ = 0.2, but no interaction with group, *F*(1,33) = 0.53, *p* > 0.47. Subsequent memory interacted with anteriority, *F*(1.28,42.19) = 4.67, *p* = 0.03, η_p_^2^ = 0.12. Separate laterality by subsequent memory ANOVA’s for each level of anteriority revealed that the SME was significant at frontal and central electrodes (both *p* < 0.01; parietal electrodes: *p* = 0.052). In further *post hoc* tests, the amplitude difference between subsequent hits and misses was larger when collapsed across the three central compared to the three parietal electrodes (*p* < 0.05), and tended to be larger when collapsed across the three frontal, compared to the three central electrodes (*p* = 0.067). Taken together, the slow wave SME was frontally distributed and present in both groups.

##### Summary

In the P300 time window only the definition condition elicited SMEs, while frontal slow wave effects were found for both groups. An unambiguous interpretation of these SMEs, however, is complicated by the apparent ERP component overlap. In particular, in the P300 time window, both a parietal positivity and a frontal negativity appeared to be prominent (**Figure 3**). To disentangle the different components, we conducted a spatio-temporal PCA.

#### Principal Component Analysis (PCA)

**FIGURE 4:**
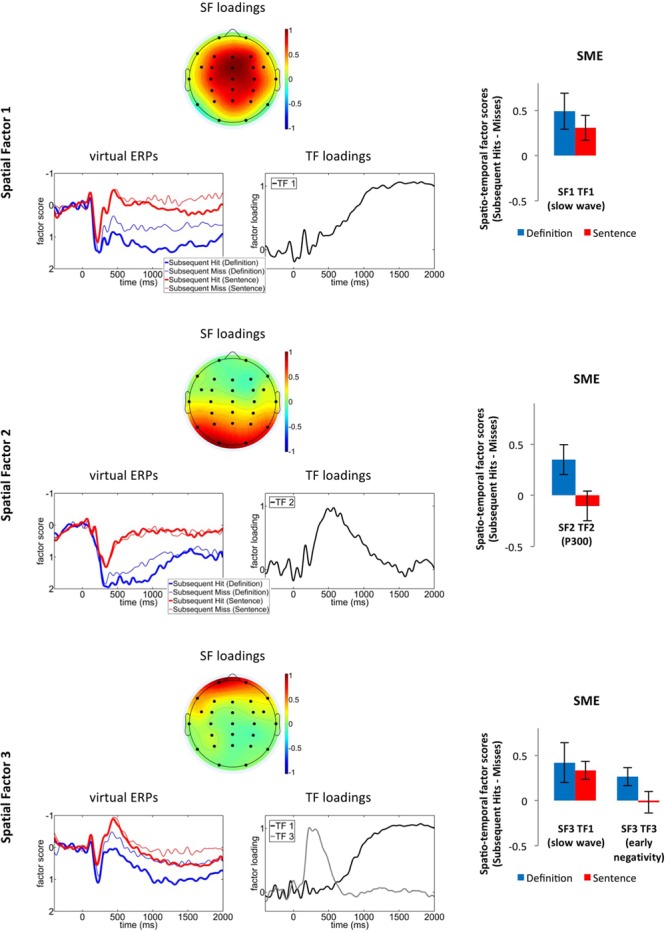
**First three spatial factors from the PCA solution: SF loadings (top panels), “virtual ERPs” for subsequent hits and misses (bottom left panels), TF loadings of those TFs that significantly varied with subsequent memory for at least one of the groups (bottom right panels), as well as the difference in corresponding spatio-temporal factor scores between subsequent hits vs. subsequent misses (i.e., the SME; right panels, error bars represent the standard error of the mean).** SF, spatial factor; TF, temporal factor.

**Figure 4** shows the spatial factor (SF) loadings, virtual ERPs, TF loadings and difference in spatio-TF scores (as measures of ERP component amplitudes) between subsequent hits and subsequent misses for the first three SFs obtained in the PCA. For the purpose of simplicity we only present results from TFs whose spatio-TF scores varied with subsequent memory. The corresponding spatio-TF scores were analyzed in 2 (subsequent memory) by 2 (group) mixed ANOVAs.

Spatial factor 1 was fronto-centrally distributed and its virtual ERPs were characterized by a pronounced positive-going slow wave. The slow wave (TF 1), exhibited more positive-going amplitudes for the definition than the sentence group, *F*(1,33) = 6.95, *p* = 0.01, η_p_^2^ = 0.17, and varied with subsequent memory, *F*(1,33) = 10.98, *p* < 0.01, η_p_^2^ = 0.25. There was no interaction between subsequent memory and group (*p* > 0.45; **Figure 4**).

Spatial factor 2 exhibited a parietal distribution similar to the distribution of the P300 component extracted by PCA in prior studies (Kamp and Donchin, 2015). We therefore concluded that this factor captures the parietal SME. Its virtual ERPs exhibited a positive peak at 400 ms (captured by TF 2), which exhibited significantly larger amplitudes for the definition- than the sentence group, TF 2: *F*(1,33) = 24.51, *p* < 0.001, η_p_^2^ = 0.43. There was also an interaction between group and subsequent memory, *F*(1,33) = 4.86, *p* = 0.04, η_p_^2^ = 0.13. *Post hoc* analyses revealed an SME in the definition, *t*(16) = 2.5, *p* = 0.03, *d* = 0.58, but not in the sentence, *t*(17) = 0.71, *p* = 0.49, group.

Spatial factor 3 exhibited a prefrontal distribution and its virtual ERPs were characterized by a negativity at 400 ms (captured by TF 3) and a slow wave (TF 1) with a similar time course as the slow wave within SF 1. The slow wave varied with subsequent memory, *F*(1,33) = 10.0, *p* < 0.01, η_p_^2^ = 0.23, and did not exhibit any main effects for- or interactions with the factor group (both *p*s > 0.29). For the early negativity (TF 3), there was a statistical tendency for an interaction between group and subsequent memory, *F*(1,33) = 3.09, *p* = 0.09, η_p_^2^ = 0.09, indicating that there was an SME in the definition, *t*(16) = 2.62, *p* = 0.02, *d* = 0.64, but not in the sentence, *t*(17) = 0.15, *p* = 0.88, group.

##### Summary

The PCA results confirm and extend the results from the mean amplitude analyses in revealing that the scalp-recorded SME in the early time window (around 400 ms) for the definition group was driven by variance in two spatio-TF combinations: SF3 TF3, capturing an early prefrontal negativity and SF2 TF2, capturing a parietal positivity. Both of these factors exhibited SMEs for the definition, but not for the sentence group. By contrast, the late frontal slow wave, which exhibited a fronto-central (SF 1) and a prefrontal (SF 3) aspect, varied with subsequent memory for both groups.

#### Between Subject Correlations

To further explore the functional significance of the fronto-central slow wave SME and the early parietal SME (i.e., those SMEs of interest to our a priori hypotheses), we calculated between-subject Pearson’s correlations among the SMEs and with recognition performance (**Table 1**). SMEs were quantified for each participant by subtracting the factor score for subsequent misses from subsequent hits. For the sentence, but not for the definition group there was a positive correlation between the frontal slow wave SME and the Pr score. Furthermore, for the definition group, a significant positive correlation was found between the parietal SME and the frontal slow wave SME (**Table 1**).

**Table 1:** Pearson’s correlation coefficients between Pr scores and event-related potential (ERP) subsequent memory effects (SMEs).

		(1) Pr score	(2) Frontal SW SME^1^	(3) Parietal SME^2^
(1) Pr score		-		
(2) Frontal SW SME^1^	*Definition*	-0.12	-	
	*Sentence*	0.50^∗^		
(3) Parietal SME^2^	*Definition*	-0.28	0.73^∗∗^	-
	*Sentence*	0.42	0.20	

*SMEs were calculated for each participant by subtracting spatio-temporal factor score for subsequent misses from subsequent hits. The first value in each cell represents the coefficient for the definition condition (*n* = 17), and the second value the one for the sentence condition (*n* = 18). ^∗^*p* < 0.05, ^∗∗^*p* < 0.01, ^1^SF1 TF1, ^2^SF2 TF2, SW, slow wave.*

## Discussion

Under otherwise matched encoding conditions and in the absence of behavioral differences in recognition performance, ERP SMEs elicited by word pairs encoded in the context of an entity defining framework (definition condition), thus promoting the formation of a single item representation (unitization), differed from those elicited by word pairs encoded in an inter-item associative encoding task (sentence condition). Our data therefore provide further evidence that these two encoding conditions are dissociable in the neuro-cognitive processes that support subsequent retrieval (Haskins et al., 2008). In particular, as predicted a late frontal slow wave SME was evident in the sentence group and an earlier parietal SME was found in the definition group only. However, not in line with our hypothesis, the definition condition additionally elicited an early frontal negativity and a late frontal slow wave SME. In the remainder of this section we discuss and extend the idea that whether the parietal or the frontal slow wave SME is observed depends on the extent to which different aspects of an experience are encoded as parts of a single, unified whole vs. encoding of an inter-item association.

### Parietal SME

Word pairs in the definition but not in the sentence condition can be unitized into a single, new concept, so item encoding benefits subsequent retrieval of the pair in the former condition only. In line with the hypothesis that the parietal SME co-occurs with item encoding, word pairs in the definition, but not in the sentence group elicited a parietal SME. We thus propose that the parietal SME reflects the encoding of rich details of a to-be-encoded unit that leads to an item representation that is distinctive from other memories and is therefore likely to be retrieved successfully on a subsequent test. Furthermore, as prior research has shown that familiarity is sufficient to retrieve the word pairs in the definition condition of this paradigm (Bader et al., 2014), a parietal SME may also indicate that the corresponding items may be subsequently retrieved on the basis of familiarity. In support of this view, in a recent analysis of the test phase ERPs of the same data as presented here, an early frontal old/new effect but no parietal old/new effect was elicited in the definition condition, while in the sentence condition the late parietal old/new effect indexing recollection was pronounced (Kamp et al., 2016).

Previous research has demonstrated the elicitation of a P300-like response in the hippocampus, whose amplitude varies with subsequent recall (Axmacher et al., 2010). Although scalp EEG data do not capture hippocampal activity directly (e.g., Knight and Scabini, 1998) and inferences from scalp ERP recordings are in most cases problematic, it is tempting to speculate that the parietal SME may co-occur with early binding processes in the hippocampus that serve the integration of multiple aspects of an experience into a unitized memory trace. This idea is tentatively supported by fMRI results from Ranganath et al. (2005), who reported a hippocampal SME for a subsequent long-term memory task only when novel, pre-experimentally unfamiliar stimuli were presented in the early portion of a delayed matching to sample task. On the other hand, hippocampal activity is not *necessary* to encode the novel units in the definition condition of the present paradigm, because this condition leads to better associative recognition than the sentence condition in patients with selective damage to the hippocampus (Quamme et al., 2007). Furthermore, fMRI evidence speaks against hippocampal involvement in the definition condition (Haskins et al., 2008; Bader et al., 2014). Further research is therefore necessary to determine the extent to which hippocampal activity contributes to the generation of the parietal SME.

Notably, the two conditions differed not only in the presence or absence of the SME, but also in the amplitudes of ERP components themselves. Therefore, due to the preceding presentation of the definition or sentence the cognitive processes evoked in the two conditions may have already differed before the word pairs were encountered. In particular, the larger parietal positivity in the definition group suggests that the definition created stronger expectancies about the upcoming word pairs, because stimuli that violate expectations typically elicit a larger P300 (Donchin, 1981). This may have facilitated preparatory processes and consequently early episodic encoding, leading to the early SMEs in the definition condition only. Nevertheless, expectancy violation is unlikely to be the crucial factor for encoding in the definition condition. If this was the case, then pairs which were judged by the participants as a difficult-to-imagine concept should have led to better subsequent recognition performance, but an analysis of this type pointed in the opposite direction. Furthermore, the parietal SME in the definition group cannot be explained solely by the fact that a larger overall P300 was elicited. If so, then within the definition group larger parietal amplitudes should be correlated with larger SMEs. There was, however, no evidence for such a correlation, *r*(15) = 0.10, *p* = 0.7. Likewise, inclusion of overall P300 amplitude for each individual as a covariate in the ANOVA analyses did not change the result patterns. This is also consistent with prior reports that the elicitation of a P300 does not guarantee that the P300 will also vary with subsequent memory (Otten and Donchin, 2000).

Taken together, the results support our hypothesis that this SME occurs during the formation of a single item representation under conditions that foster unitization encoding.

### Early Frontal SME

In the same time window as the early parietal SME a prefrontal ERP component with a negative polarity also varied with subsequent memory. As this SME was also observed only in the definition group, its contribution to episodic encoding may lie within some aspect of integrating multiple items into a unified whole, such as semantic processes elicited during unitization of word pairs. One possibility is that, together with the parietal SME, it reflects coupling of prefrontal and medial temporal areas during the early unitization, or item encoding process. For example, Haskins et al. (2008) reported a SME in the inferior frontal gyrus in the definition condition and proposed that it reflects controlled retrieval of associations of the word pair that aids formation of the new concept. Furthermore, Ranganath et al. (2005) reported a SME in DLPFC to co-occur with the hippocampal SME during the early period of their delayed matching to sample task. Alternatively, the modulation of the frontal negativity during encoding in the definition condition could reflect activity in the PrC, as a PrC SME has also been reported for the definition condition (Haskins et al., 2008). Unfortunately, the spatial resolution of ERPs is too low to allow us to arbitrate between these alternatives. Follow-up studies could, however, use source localization techniques to identify likely sources for the observed effect.

The latency and negative polarity of the early frontal SME is also consistent with the N400-like response that has been reported in the anterior MTL (McCarthy et al., 1995), whose amplitude has also been shown to vary with subsequent recall for verbal study materials (Fernández et al., 1999). Particularly, this SME is observed for high-frequency words only, whose lexical representations are easier to access and exhibit richer semantic associates than low-frequency words (Fernández et al., 2002). The early frontal negativity in our study may reflect functionally similar brain activity, which also supports the idea that it indexes semantic integration processes that accompany the formation of novel conceptual units.

### Frontal Slow Wave SME

We predicted a double dissociation of the parietal and the frontal slow wave SME: On the one hand, as we confirmed in our dataset, the parietal SME should be elicited only when the to-be-encoded word pair is encoded as a single unit. However, we also predicted that the integration of multiple discrete items into a flexible inter-item associative representation rather than the encoding of a single, unitized item are reflected in the frontal slow wave, so the sentence- but not the definition condition should elicit this SME. Disconfirming the latter prediction, in both groups, both the prefrontal and the fronto-central aspect of the frontal slow wave were more positive-going for word pairs associated with subsequent hits than misses, so the predicted double dissociation was not evident.

The division of slow wave activity into the fronto-central and the prefrontal aspect warrants some discussion. Both the time course and the variance with subsequent memory were remarkably similar between the two slow wave PCA factors. Furthermore, participants who exhibited a strong prefrontal slow wave SME also exhibited a strong fronto-central SME [*r*(35) = 0.704, *p* < 0.001, across participants in both groups], suggesting that the SMEs may reflect brain activity in a network of areas that contribute to similar aspects of word pair encoding. Perhaps more importantly, we neither had a priori hypotheses about different kinds of frontal slow wave SMEs, nor do our results provide a way to functionally dissociate them. Therefore, in the following we will assume that they do not reflect distinct cognitive processes. Nevertheless, whether slow wave SMEs with prefrontal and fronto-central distributions can be functionally dissociated remains an open question that must be addressed in future research.

To interpret the frontal slow wave SME, it is useful to first consider the correlational nature of SMEs: They reflect brain activity that *co-occurs* with successful encoding, so they can reflect processes that play a *critical role* in enhancing the study information’s memorability (which would be the case if participants relied on the outcome of this process during retrieval), or processes that are *a consequence of* the processes that enhance memorability^2^. If the frontal slow wave SME is of the former type, its magnitude should correlate between subjects with performance on the recognition test. Such a correlation was evident in the sentence group only. To follow the principle of parsimony, we are hesitant to conclude that frontal slow waves in the two conditions reflect entirely different cognitive processes. Rather, our experimental conditions appear to differ in the extent to which participants rely on the mnemonic outcome of these brain processes during subsequent retrieval. This is in line with prior findings that retrieval in the definition condition relies less on recollection, a process that is particularly important to retrieve inter-item associations (Yonelinas, 2002), than in the sentence condition (Bader et al., 2010, 2014; Haskins et al., 2008; Kamp et al., 2016).

Another clue to unravel the functional role of the frontal slow wave SME in the definition condition is the correlation of its magnitude with the parietal SME. The different scalp distributions (supported by the SMEs being captured in different PCA factors) exclude the possibility that the frontal slow wave SME is merely a continuation of the parietal SME. Rather, the dependency between the two effects in our definition group suggests that successful early unit encoding (reflected in the parietal SME) was followed by further working memory-based elaboration (reflected in the frontal slow wave). Perhaps the lack of significant positive correlations between the frontal slow wave SME and memory performance in the definition group can be explained such that encoding relied on a cascade of activity in a broader network (supported by the SMEs in multiple ERP components). Each individual SME may therefore not have been as diagnostic for subsequent memory performance as the frontal slow wave SME in the sentence group.

Also worth considering is the prior finding of a larger parietal old/new effect for correctly remembered word pairs in the sentence than in the definition condition (Bader et al., 2010; Kamp et al., 2016). This suggests that recollection plays a larger role in the sentence condition. Unlike what one may predict from the literature review presented in the introduction section, there is therefore no direct correspondence of the frontal slow wave SME to subsequent recollection-based retrieval.

Hence we propose that the frontal slow wave SME may reflect working memory processes that support the encoding of item pairs regardless of the encoding strategy engaged and regardless of the extent to which subsequent retrieval is based on recollection or familiarity. Notably, in any encoding situation associations are formed to some extent with objects that have occurred in the same episodic context, and between items and the encoding context or previous knowledge. The frontal slow wave may thus not be an index of inter-item encoding *per se*, but of any situation in which episodic encoding gains memorability for the subsequent test from active binding processes. This is in line with reports of frontal slow wave SMEs for individual items, such as when words are encoded and subsequently tested in a standard old/new recognition test (e.g., Bauch and Otten, 2012). The resolution of this issue has to await further research, because in the present study we did not manipulate or measure the extent to which other forms of episodic information were bound and subsequently retrieved.

### Conclusions and Future Directions

Our results demonstrate that in the absence of behavioral differences in memory performance, the ERP components that vary in amplitude with subsequent retrieval success depend on the nature of the encoding task. Successful encoding of unitized items is associated with the parietal SME, which during formation of a novel concept from discrete items may interact with semantic integration processes implemented in the prefrontal cortex and/or the PrC. A frontal slow wave, by contrast, appears to co-occur with processes more generally involved in associative encoding. We therefore propose that ERP SMEs are not as unreliable as some have suggested (Johnson, 1995). Rather, different parameters, such as encoding strategies that support the formation of inter-item relations vs. strategies that lead to a coherent entity in memory, introduce variance that results in different kinds of ERP SMEs between studies. In the long run, an understanding of the eliciting conditions of different kinds of SMEs may aid investigations of the mechanisms contributing to episodic memory processes under specific circumstances, for example in clinical subject populations.

## Materials and Methods

The methods were in line with the declaration of Helsinki and were approved by the local ethics committee. All participants provided informed consent before the experiment.

### Participants

Forty-two German native speakers were paid eight Euros per hour and were assigned randomly to one of the two conditions. Four participants in the definition- and three participants in the sentence group were excluded because less than 10 artifact-free trials were available for one of the ERP averages of interest. The remaining participants were on average 21.67 (*SD* = 2.44) and 23.61 (*SD* = 3.07) years old in the definition (*n* = 17; 9 males) and sentence (*n* = 18; 7 males) group, respectively. There was no age difference between participants in the two groups (*p* > 0.70).

### Stimuli

We used a stimulus set that has been developed for a previous study (Bader et al., 2014)^3^, and includes 170 word pairs that can be combined into novel, grammatically legal compound words. According to a pilot rating study conducted by Bader et al. (2014) the two words of a pair were not semantically related. Each pair was associated with (1) a definition that framed the compound word as a new concept and (2) a sentence that included two blanks in which the two words could be inserted (**Figure 1**; **Table 2**). In the sentence condition, the word pairs were thus treated as two discrete items (inter-item encoding) while in the definition condition they could be unitized into a single compound (unitization or item encoding). Words which were parts of the pairs did not reappear in the definitions or sentences.

**Table 2:** Example stimuli from the experiment.

Word pair	Definition	Sentence
Atom, apartment	Housing, which is very small	The ___ was exhibited in the ___.
Blood, sky	A blanket of clouds that is stained in red	The ___ splashed into the ___.
Window, rock	A chunk that lies on the ledge	The ___ was hit by the ___.
Village, boot	A foot garment that is worn in rural areas	In the ___, people liked to wear ___.
Iron, tongue	A flap that is made of metal	The ___ pierced her ___.

*Stimuli have been translated from German to English.*

In the test phase 80 word pairs were presented in the identical pairing as during encoding, and 80 word pairs were presented in a new combination. To create the recombined pairs, 80 words that were presented on the left side of a pair at encoding were combined with a word that was presented on the right side of a different pair. Pairs to be presented as old vs. recombined were randomly selected for each participant. The sentences/definitions were not presented again during the recognition test.

All stimuli were presented in white Arial font on a black background. Word pairs were presented in font size 32, and definitions/sentences in font size 22. Between the two words of a pair four blank spaces were inserted. Together, word pairs spanned up to 9° of visual angle.

### Procedure

The session began with the preparations for the EEG recording, which took about 30–45 min. Then, participants were seated in front of a computer screen in an electrically shielded, sound attenuated room. The experiment was presented using E-prime 2.0 software and participants used a serial response box to provide their answers.

#### Encoding

The instructions were modeled after Bader et al. (2010). Participants assigned to the definition condition were instructed that they would be presented with new concepts that consisted of two words that could be combined into a compound, together with a definition that described the meaning of the new concept. Their task was to try to imagine this new concept and judge its plausibility. Participants in the sentence condition were told that they would see a sentence with two blanks, followed by a word pair. They were to mentally insert the words into the blanks, try to imagine the resulting new sentence, and rate its plausibility (**Figure 1**). In both conditions the instructions emphasized that there was no correct answer, but that the participants should rely on their personal sense of the plausibility of the new concept/sentence. No mention of the subsequent recognition test was made.

Participants completed 4 practice trials while the experimenter was present to answer any clarification questions, followed by 160 experimental trials. At the end of the encoding phase, 6 additional trials were inserted, which were used to generate the practice pairs for the test phase and were not further analyzed. Trial order was random and there were three self-paced breaks.

##### Trial Structure

The trial structure in the encoding phase is illustrated in **Figure 1**. The sentence or definition was first shown horizontally centered and 6 cm below the center of the screen. After 4 s, in addition a fixation cross was shown for 1 s in the center of the screen, which participants were instructed to fixate as soon as it appeared. Next, the fixation cross was replaced by the word pair for 2 s. The definition/sentence remained on the screen up to this point.

After a 500 ms blank screen, participants were prompted to provide their rating of how well they could imagine the new concept/sentence on a scale of 1 (“very well”) to 2 (“rather well”) to 3 (“rather poorly”) to 4 (“very poorly”; the scale was reversed for half of the participants). The button press terminated the screen. The 2 s long inter-trial interval consisted of a blank screen.

#### Recognition

After a 5 min long distraction task (counting backward in steps of 3), the recognition test began, which was identical in the two conditions. The instructions explained that participants would be presented with word pairs, some of which were seen in the same combination as in the encoding phase. These were to be evaluated as “old.” Others would consist of two previously studied words in a novel combination, and the correct answer to such pairs was “new” (note that in the present article we refer to these pairs as “recombined”). Participants first completed six practice trials, which were constructed from the final six encoding trials and any remaining questions were answered. The experimental trials were presented in pseudorandom order, with the restriction that no more than five pairs of the same type (old or recombined) were presented sequentially. There were three self-paced breaks.

##### Trial Structure

A word pair was first shown for 2 s. After a blank screen (500 ms), participants were prompted to provide their answer on a scale of 1 (“definitely old”) to 2 (“probably old”) to 3 (“maybe old”) to 4 (“maybe new”) to 5 (“probably new”) to 6 (“definitely new”; the rating scale was reversed for half of the sample). The response terminated the screen. Between two recognition trials, a fixation cross was shown for 2 s.

### Behavioral Data

To analyze the ratings given at encoding for each condition, we calculated the proportion of trials that had received a “very well” or “rather well” plausibility rating. Secondly, to quantify associative recognition performance, we calculated Pr scores (hits to old pairs – false alarms to recombined pairs; collapsed across all plausibility ratings), (1) by collapsing across confidence levels (“definitely old,” “probably old,” and “maybe old”), and (2) by only considering high confidence responses (“definitely old”) as hits and false alarms. The latter analysis was included because it paralleled the subsequent memory analyses (see below). Behavioral measures were compared between the groups using independent samples *t*-tests.

### EEG Recording and Analysis

The EEG was recorded from 28 Ag/AgCl scalp electrodes embedded in an elastic cap according to the 10–20 electrode system (Fp1, Fp2, F7, F3, Fz, F4, F8, FC5, FC3, FCz, FC4, FC6, T7, C3, Cz, C4, T8, CP3, CPz, CP4, P7, P3, Pz, P4, P8, O1, O2, and A2) and on-line referenced to the left mastoid electrode (A1). Ocular activity was recorded from four electrodes placed at the outer canthi of each eye and above and below the right eye, and FCz was used as the ground electrode. Impedances were kept below 5 kΩ. The EEG was amplified with BrainAmp (Brain Products, Inc.) DC amplifiers from DC to 250 Hz with an analog 50 Hz notch filter, and digitized at 500 Hz.

For the off-line ERP analysis we used Brain Vision Analyzer software. The data were first bandpass-filtered at 0.1–20 Hz and re-referenced to linked mastoids. Next, we extracted segments from the study phase, which included an interval of 400 ms before to 2000 ms after word pair onset. The segments were corrected for eye blinks and saccades using a regression-based method (Gratton et al., 1983). Trials containing amplitude steps of 20 μV/ms were considered artifactual and excluded from further analysis. Finally, separate subject ERP averages were calculated for trials associated with subsequent high confidence hits (from now on referred to as “subsequent hits”) and trials associated with low confidence hits or misses (“subsequent misses”). The division according to this criterion was necessary to obtain enough trials in each category. It is worth noting that considering only high confidence hits as “subsequent hits” leads to a focus on subsequent recollection. The subject averages were baseline corrected using the average amplitude of the 400 ms preceding the word pair.

#### Mean Amplitudes

We report mean amplitude measures in two time windows. The first (300–600 ms after pair onset) time window was centered around the P300 peak and comprises a typical time window for the parietal SME. The remainder of the encoding trial (600–2000 ms) was initially analyzed in successive 200 ms long time windows. The result patterns were equivalent for all time windows between 600 and 1200 ms and were similar to those of the P300 window, so for the purpose of conciseness we will not report these results. Furthermore, result patterns were equivalent for all time windows between 1200 and 2000 ms, so we collapsed across this second time window to quantify the frontal slow wave.

#### Principal Component Analysis (PCA)

Visual inspection of the ERP waveforms suggested that variance from more than one ERP component contributed to the scalp-recorded subsequent memory- and between group variance in the P300 time window. Such temporally overlapping components cannot be disentangled by using simple mean amplitude analyses. To this end, in an additional analysis step the subject averages for all electrodes and for subsequent hits and subsequent misses were submitted to a spatio-temporal PCA (Spencer et al., 1999) using the Matlab-based ERP PCA toolkit (Dien, 2010a). For both PCA steps, we used Promax rotation without the Kaiser normalization option (for a detailed discussion, see Dien, 2010b). In the spatial step, 8 SFs were rotated, accounting for 94% of the total variance in the data. For each SF, we calculated “virtual ERPs,” which are plots of SF scores over time and can be conceptualized as an index of the specific SF’s activity for each time point (Spencer et al., 1999). SFs 1 (variance accounted for after rotation: 45%) and 2 (19%) exhibited the spatial distribution of the frontal slow wave and the parietal SMEs, respectively, and the corresponding virtual ERPs exhibited temporal characteristics typical of the respective ERP component. Furthermore, SF 3 (9%) captured a prefrontal negativity that temporally overlapped with the parietal SME and its virtual ERPs also appeared to vary with subsequent memory. Because SF4 did not capture encoding-related activity and did not exhibit spatio-temporal characteristics of SMEs we had a priori hypotheses for, we restricted all further analyses to these first three SFs. In the next step, we rotated, for each SF, six TFs, accounting for a total of at least 90% of the variance of the SF. The spatio-TF scores of the combinations of SF and TF that were of interest to our hypotheses due to their resemblance of the ERP components of interest were analyzed as measures of ERP component amplitudes in the statistical tests.

#### Statistical Analysis

All statistical analyses were conducted using IBM SPSS software. We first analyzed mean amplitudes from nine electrodes (F3, Fz, F4, C3, Cz, C4, P3, Pz, and P4) in three (anteriority: frontal, central, and parietal) by three (laterality: left, center, right) by two (subsequent memory: subsequent hit vs. subsequent miss) by two (group: sentence vs. definition) mixed ANOVAs. Whenever the assumption of sphericity was violated, we report Greenhouse-Geisser corrected degrees of freedom and *p*-values. Spatio-TF scores obtained in the PCA were analyzed in two (subsequent memory) by two (group) mixed ANOVAs. Significant interactions were followed up by lower level ANOVAs and *t*-tests. As measures of effect size, we report partial eta squared (η_p_^2^) for ANOVA results, and Cohen’s *d* for *t*-tests.

## Ethics Statement

The study was approved by the ethics committee of faculty 5, Saarland University. All participants were provided with written and oral information about the experiment before their participation and provided written informed consent.

## Author Contributions

S-MK, RB, and AM participated in study design, discussion of the data and writing of the manuscript. S-MK led data collection and analysis.

## Conflict of Interest Statement

The authors declare that the research was conducted in the absence of any commercial or financial relationships that could be construed as a potential conflict of interest.

## Footnotes

1 A series of additional behavioral analyses (which we do not report here for the purpose of conciseness, but which will be reported elsewhere) also did not reveal evidence for any differences between the two conditions. This included a separate analysis of performance for trials rated as highly and poorly plausible, a receiver-operating characteristics analysis, as well as separate analyses for hits and false alarms at all confidence levels.

2 Another option (which is, however, not relevant for the present argument) is that SMEs may be a by-product of successful encoding.

3 In this prior study, some words were converted to the plural form in order for the “reversed” combination of each pair to be a legal compound word as well. Since in the present study we did not use reversed pairs, we used the singular form wherever possible. That is, the plural form was only used when it was necessary in order for the two words to form a legal compound word in German.
